# Supplementary material for: MISATO: machine learning dataset of protein–ligand complexes for structure-based drug discovery
Source: Nat Comput Sci. 2024 May 10;4(5):367–78. doi: 10.1038/s43588-024-00627-2 (PMC11136668; doi:10.1038/s43588-024-00627-2)
Supplement: Supplementary file 1 — Supplementary Tables 1–5, Figs. 1–15 and Sections 1–7. [file 43588_2024_627_MOESM1_ESM.pdf]

nature computational science

Resource

https://doi.org/10.1038/s43588-024-00627-2

MISATO: machine learning dataset of protein–ligand complexes for structure-based drug discovery

In the format provided by the authors and unedited

1. Figures and Tables

Supplementary Table 1: Overview of second affinity benchmark.
Details of the curated benchmark for a binding affinity task (Fig. 6 main text). Experimental techniques comprise surface plasmon resonance (SPR), scintillation proximity assay (SPA), radioactive filter binding assay (RFBA), Rapidfire assay, and isothermal titration calorimetry (ITC).

Set	1	2	3	4	5
Number of ligands	18	42	15	33	19
Protein	SH2	sEH	Kinase	Farnesoid-x-receptor	Trm
Affinity type	KD	IC50	KD	IC50	KD
Range in affinity [kcal/mol]	11.3	6.9	5.3	6.2	4.0
Technique	SPR, SPA	Rapidfire	RFBA	SPA	ITC
Example PDB-id	1O43	5AI0	5NJZ	5Q0I	6QQT

Supplementary Table 2: Calculated properties for QM and MD.

Details of the calculated QM properties for the ligand (left panel) and the MD properties on the dynamic traces (right panel).

Ligand	Protein-ligand dynamics
Mulliken charges (AM1, PM6, GFN2-xTB, GFN2-xTB/water, GFN2-xTB/wet octanol)	MMGBSA interaction energy
AM1-CMx charges (x=1,2,3).	buried SASA
Atomic and molecular D4-polarizabilities (in gas, water and wet octanol)	COM-distance
Curated bond orders and atomic hybrizidations	rmsd complex
Molecular electronegativities	rmsd ligand
Molecular electron affinities	
Molecular ionization potentials (including Koopman)	
Molecular hardness	
Orbital and charge-based reactivity (Fukui) indices for electrophilicity, nucleophilicity and radical behavior	
Orbital and charge-based atomic softnesses with respect to electrophiles, nucleophiles and radicals	
static logP	
Electronic densities	

Supplementary Table 3: Overview of second affinity benchmark.

Details of the second benchmark used for the evaluation of Supplementary Figure 4.

Set	1	2	3	4	5	6
Number of ligands	342	54	82	44	82	40
Proteins	Kinase	CDK2	Epoxide hydrolase	Src kinase	BTK	MetRS
Range in affinity [kcal/mol]	13.12	13.47	14.83	9.64	11.18	7.13
Example PDB-id	1AQ1	1H1P	1ZD2	2HWO	3GEN	4EG5

Supplementary Table 4: Links to access resources for MISATO.

Overview of resources across different platforms provided by the MISATO database.

Resource	Platform	Link
Repository including instructions to access the dataset and apply the AI models.	Github	https://github.com/t7morgen/misato-dataset
The dataset is provided via Zenodo and contains the QM, MD, electronic densities, and MD restart files.	Zenodo	https://zenodo.org/record/7711953
We recommend using our container images to analyze and run AI models on the dataset.	Docker-hub	https://hub.docker.com/r/sab148/misato-dataset
Integration of MISATO with example applications and demos.	Hugging Face	https://huggingface.co/MISATO-dataset

Supplementary Table 5: Splits of QM and adaptability model.

Number of samples in train, validation and test for the QM and adaptability AI models presented in the paper.

Task	Number of Samples		
	Train	Val	Test
QM	15506	1939	1938
MD	13765	1595	1612

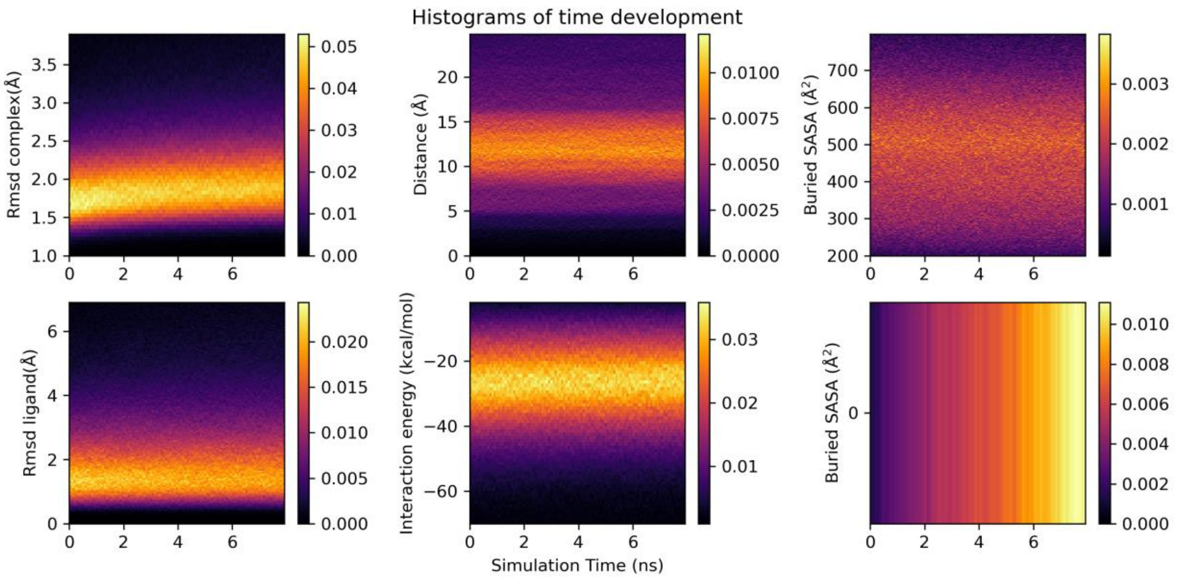

Supplementary Figure 1: Overview of the MD derived properties.

Heatmaps of the histograms of different properties of all protein-ligand complexes for each recorded timestep. The different colors represent different probabilities of a given bin from yellow (high probability) to magenta (low probability). In general, the calculated properties are quite stable through the simulation, which is a good indication that a converged ensemble is captured. Nevertheless, for individual cases the RMSD increases within the simulation time due to a conformational rearrangement of the structure.

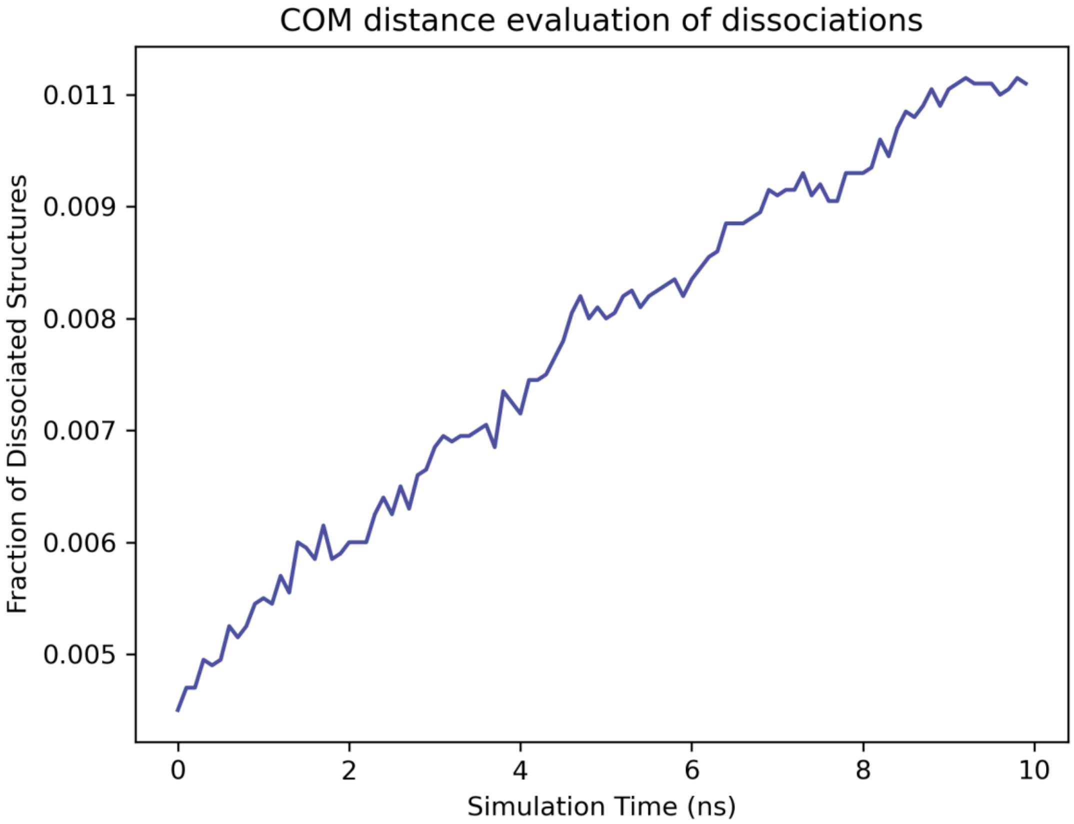

Supplementary Figure 2: Fraction of dissociated structures in the MD simulations. The simulation time in ns is shown against fraction of dissociated structures (see also Supplementary Figure 1, lower right panel). We defined a simulation state as dissociated if the COM distance between ligand and receptor was 5 Å higher in the given snapshot than for the crystal structure. With increasing simulation time, the fraction of dissociated structures increases. The simulation of an entire binding event (unbinding and reassociation) is not possible within 10 ns simulation time, so that only the dissociation of the ligand from the molecule was observed. Overall, a quite low number of 183 dissociation events was tracked, which corresponds to around 0.1 % of the simulations.

Supplemental Figure NMR characterization of m7GTP interaction with pb2

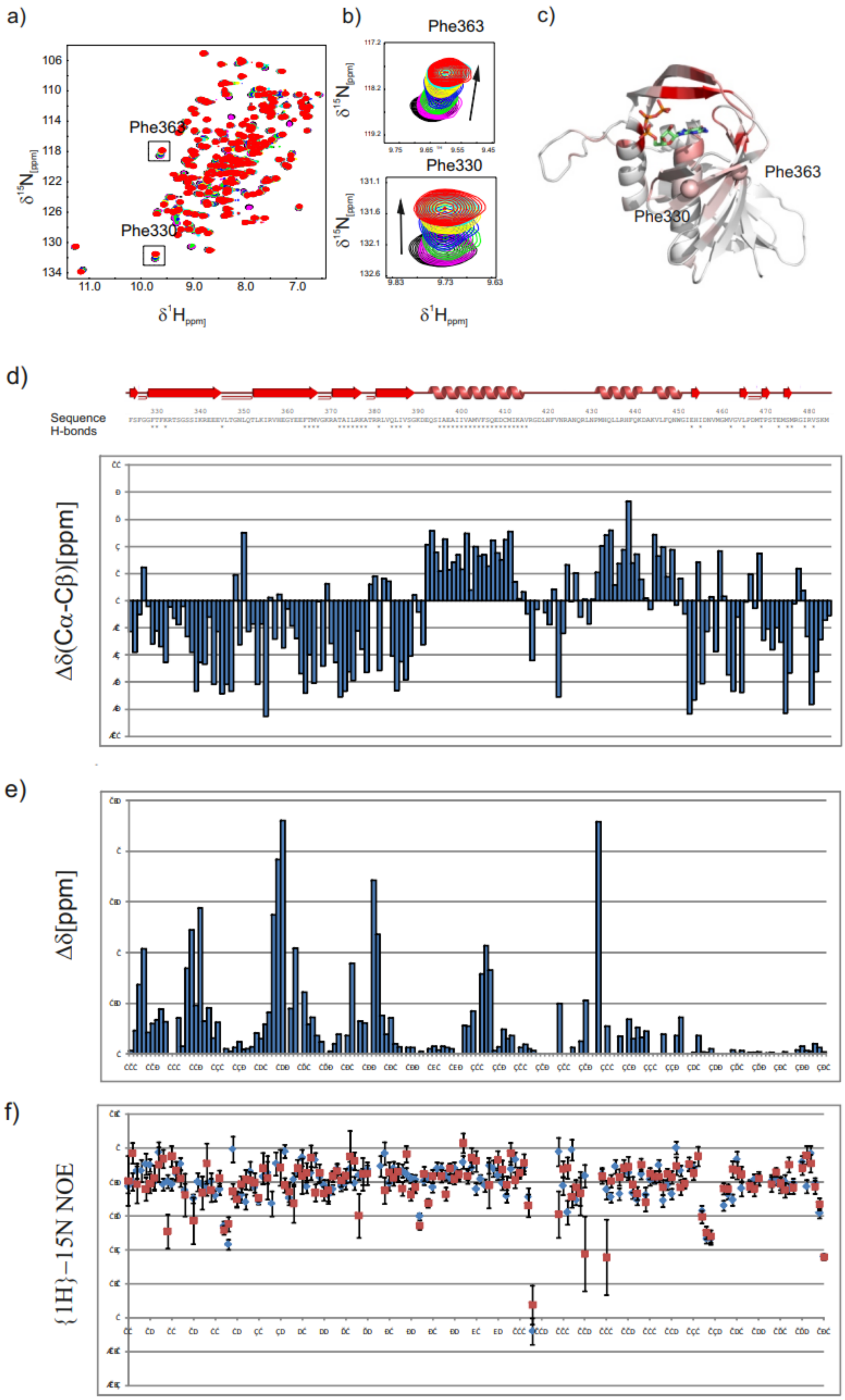

Supplementary Figure 3: NMR characterization of m7GTP interaction with pb2.

a) $^{1}H,^{15}N$-HSQC spectra of PB2 cap binding domain interacting with $m^{7}GTP$ at different protein:ligand molar ratio, (black 1:0, magenta 1:0.2, green 1:0.4, blue 1:0.6, yellow 1:0.8, cyan 1:1, red 1:1.4). b) Zoomed view of two residues showing binding in fast exchange on the NMR chemical shift time scale. c) PB2 crystal structure bound to m7GTP (PDB 2VQZ, {Guilligay, 2008 #231}) with backbone coloured by a gradient from white to red for residues with increasing chemical shift perturbations after addition saturating amounts of $m^{7}GTP$ (1.4 mol equivalents). Amide protons of the two residues for which the spectral changes are monitored are shown as spheres on the left. The binding affinity to the $m^{7}GTP$ ligand was estimated from NMR titrations and corresponds to a dissociation constant in the low micromolar range, K_D = 1.5 ± 1 mM. The chemical shift perturbations observed during the NMR titration are consistent with the binding site observed in the $m^{7}GTP$ bound crystal structure.

d) NMR ^{13}C secondary chemical shifts are displayed on the primary sequence. Slowly exchanging backbone amides indicating hydrogen bonding are indicated by "*".

e) Chemical shift changes (Dd = $\{(Dd\ (^{1}H)^{2})*10+ Dd(^{15}N)\}^{1/2}$) comparing the free and $m^{7}GTP$-bound PB2 cap binding domain.

f) ^{15}N relaxation analysis of PB2 alone (blue) and bound to $m^{7}GTP$ (red). $\{^{1}H\}$-^{15}N heteronuclear NOE. No signals were observed for residues 104-107 in both the apo and m7GTP-bound form, presumably to exchange broadening. NMR chemical shifts and ^{15}N relaxation data indicate two flexible loops, the 425-loop, between a2 and a3, and the 459-loop, between b8 and b9. Data for other residues could not be analysed to due spectral overlap. Relaxation data indicates that both the apo and cap-bound protein tumble as monomers in solution with a correlation time of t_c≈13ns. The data fitting including standard deviation error bars are generated by NMRView. The experimental uncertainties in the relaxation's spectra were propagated to the exponential curve fitting for error calculations.

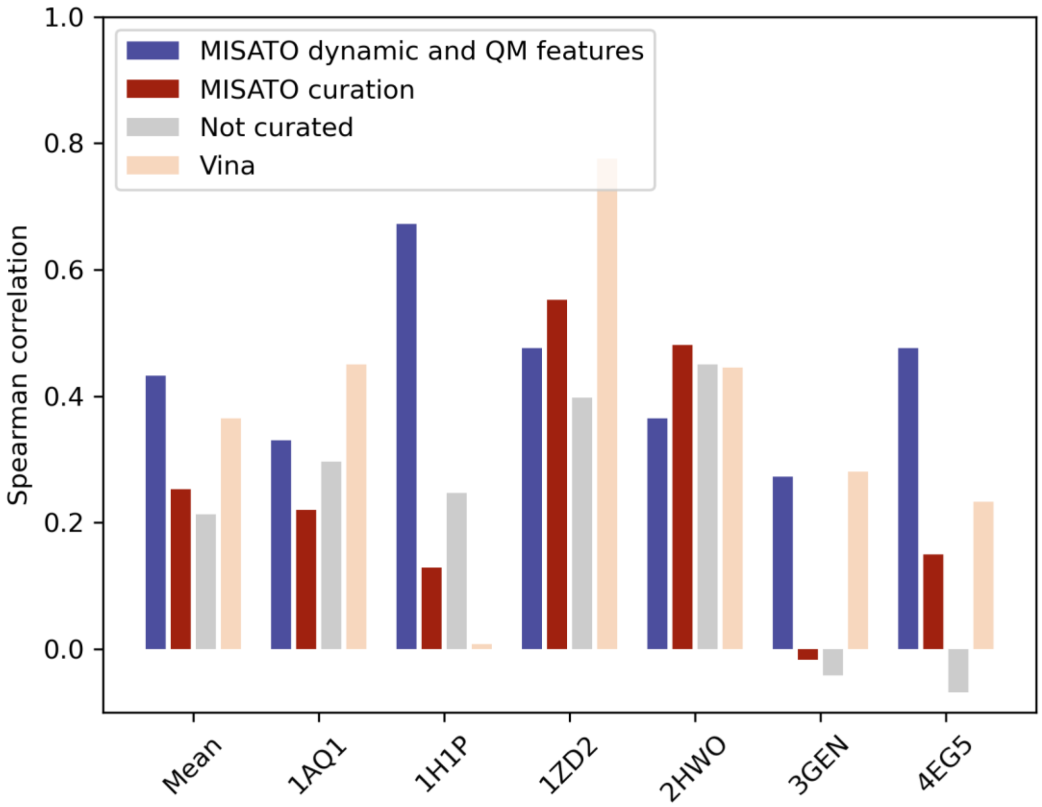

Supplementary Figure 4: Results for the binding affinity model on a second benchmark set.

Figure as Fig. 6a (main text) but for a different benchmark set. For this benchmark set we chose the six biggest clusters of protein structures (identified through the UniProt identifier). This benchmark is considered harder than the first because the affinity data comprises differing experimental techniques within one set and originates from different publications. Moreover, the sets contain on average a higher number of data points than in the first benchmark. The MISATO affinity including dynamic and QM features (0.43) has a clear improvement in the correlation over the other methods (MISATO curation 0.25, not curated 0.21, Vina 0.37).

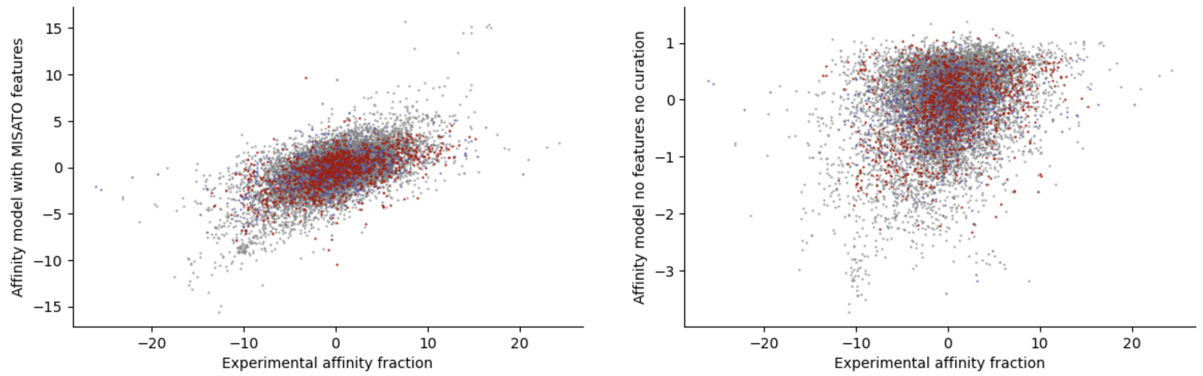

Supplementary Figure 5: Comparison of binding affinity model across the splits. The affinity model was compared on all structures with (left panel) and without (right panel) adaptability and QM features and curation to the corresponding experimental values along the different splits. The correlations to the experimental values are quite similar for each of the splits. Including MISATO features was consistently better (train (grey, 0.52), validation (blue, 0.43), test (red, 0.49), holdout (red, 0.59)) than without the features (train (grey, 0.23), validation (blue, 0.16), test (red, 0.22), holdout (red, 0.38)).

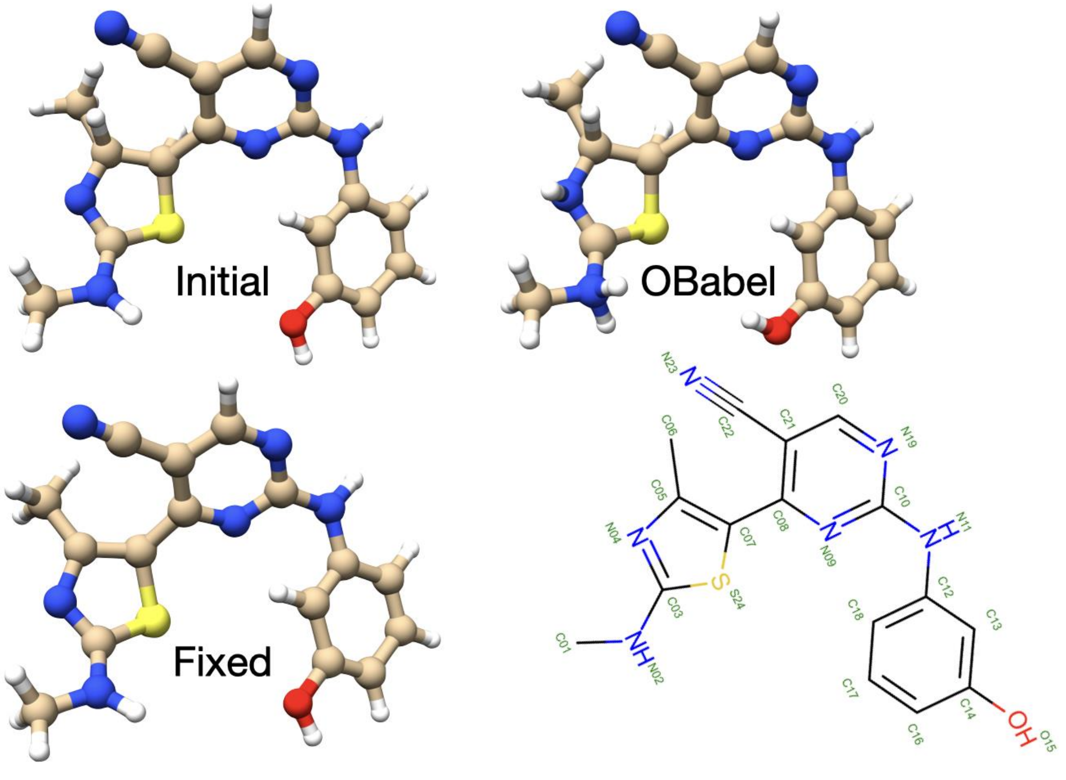

Supplementary Figure 6: Structure of 4BCN.

Initial, Openbabel and fixed structure for 4BCN. The two-dimensional representation of the structure reported in the PDB database is also given [put consistent 2D representations]. Color and character keys: Nitrogen (N, blue), Sulfur (S, yellow), Oxygen (O, red), Carbon (C, beige), Hydrogen (H, white).

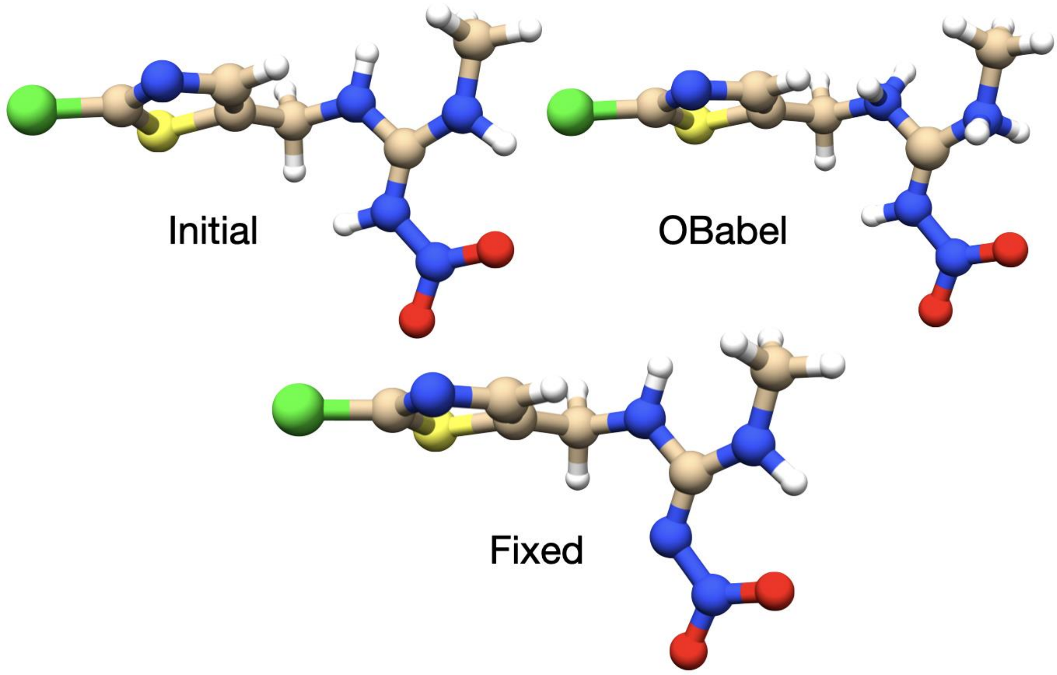

Supplementary Figure 7: Structure of 2ZJV.

Initial, Openbabel and fixed structure for 2ZJV. Note that that it is highly unlikely that the nitrated nitrogen atom is protonated, due to mesomeric effects from the nitro group. Color and character keys: Nitrogen (N, blue), Sulfur (S, yellow), Oxygen (O, red), Carbon (C, beige), Hydrogen (H, white), Chlorine (Cl, green).

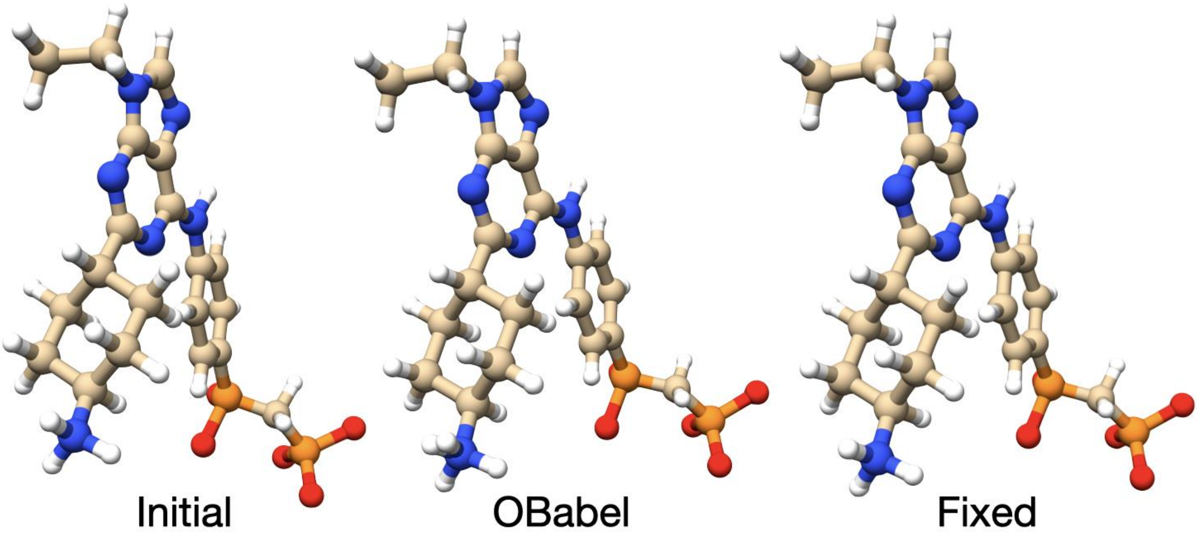

Supplementary Figure 8: Structure of 2BDF.

Initial, Openbabel and fixed structure for 2BDF. The lowest nitrogen atom (from amino group) is in explicit violation of the octet rule. Furthermore, there are two overlapping protons. Color and character keys: Nitrogen (N, blue), Sulfur (S, yellow), Oxygen (O, red), Carbon (C, beige), Hydrogen (H, white), Phosphor (P, orange).

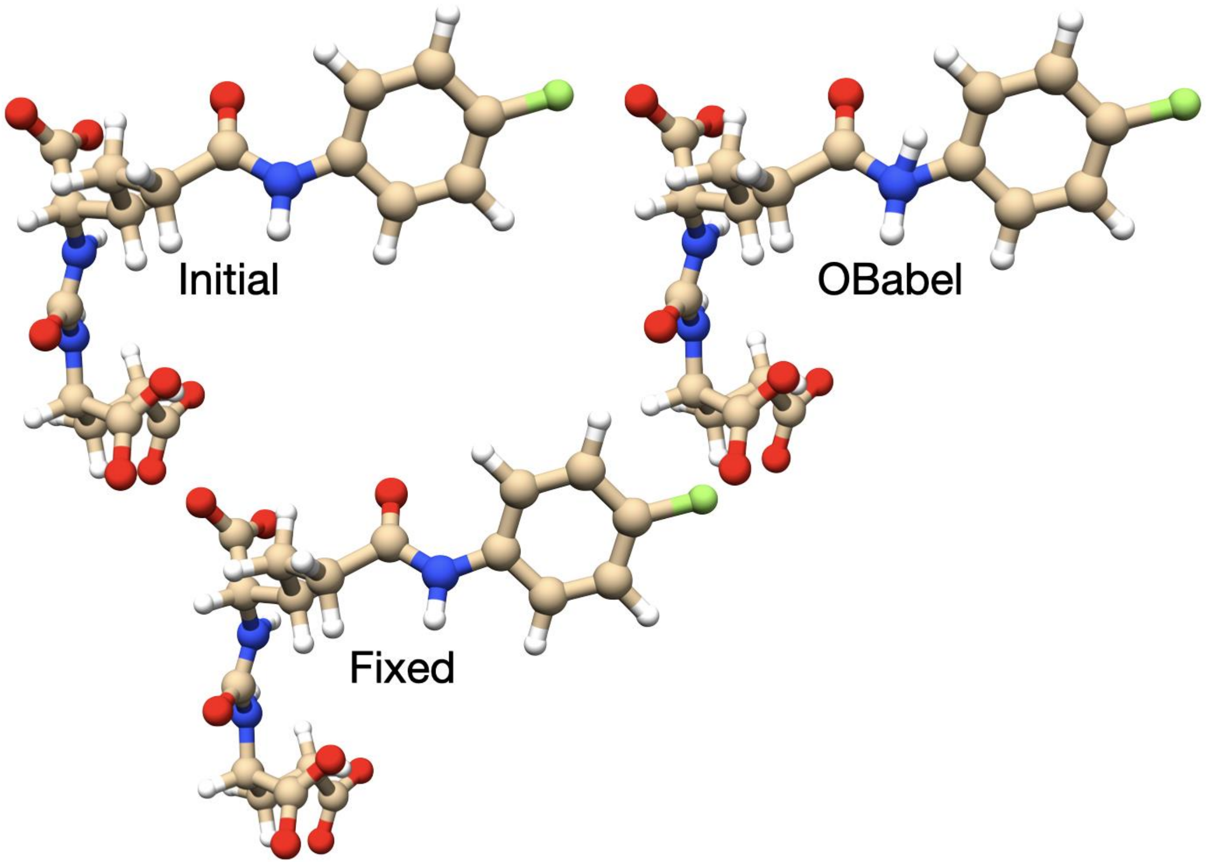

Supplementary Figure 9: Structure of 6H7Y.

Initial, Openbabel and fixed structure for 6H7Y. Color and character keys: Nitrogen (N, blue), Sulfur (S, yellow), Oxygen (O, red), Carbon (C, beige), Hydrogen (H, white), Fluorine (F, light green).

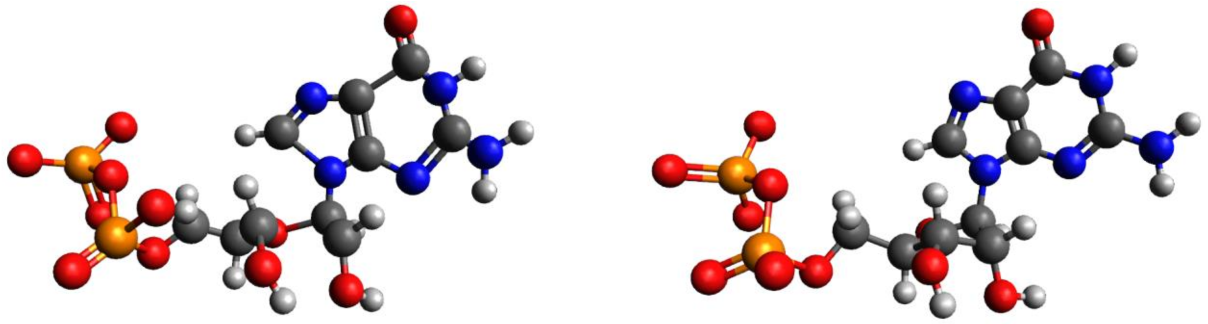

Supplementary Figure 10: Structure of 3ZY2.

Original and fixed structure of the ligand 3ZY2 using Avogadro's UFF structure optimization tool. Color and character keys: Nitrogen (N, blue), Sulfur (S, yellow), Oxygen (O, red), Carbon (C, black), Hydrogen (H, white), Phosphor (P, orange).

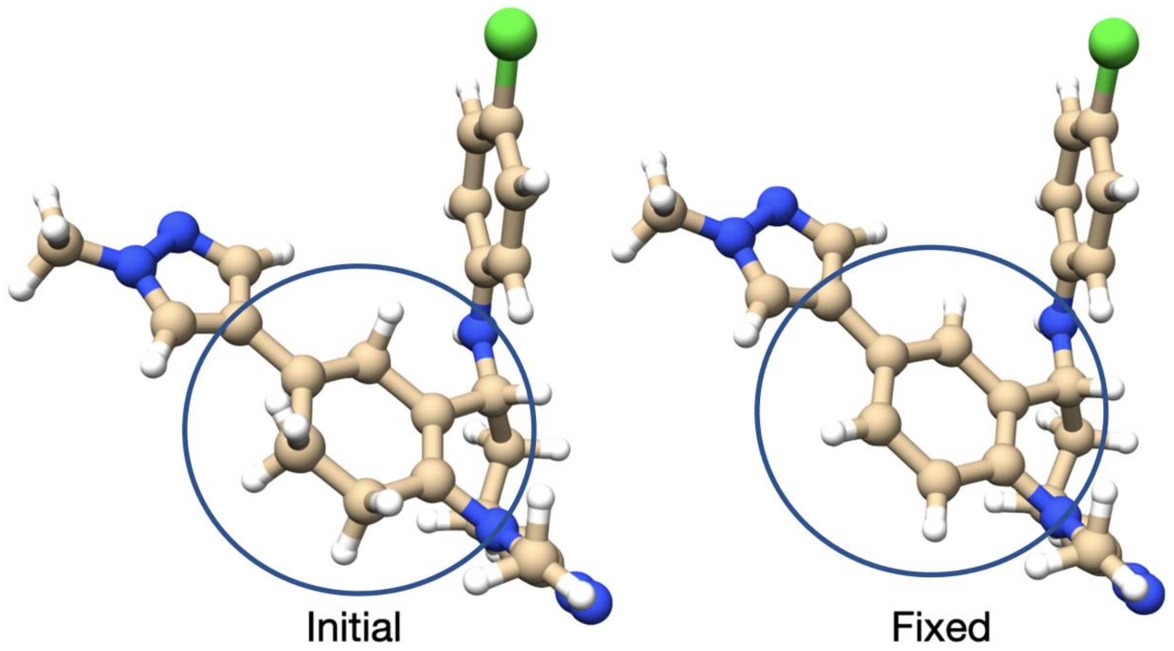

Supplementary Figure 11: Structure of 6K05.

Original and fixed structure for the ligand in 6K05. The important atoms are marked by a circle. Inconsistencies in the geometries were identified using population analysis, which allowed us to further identify protonation states inconsistent with a given atomic hybridization. Nitrogen (N, blue), Oxygen (O, red), Carbon (C, beige), Hydrogen (H, white), Chlorine (Cl, green).

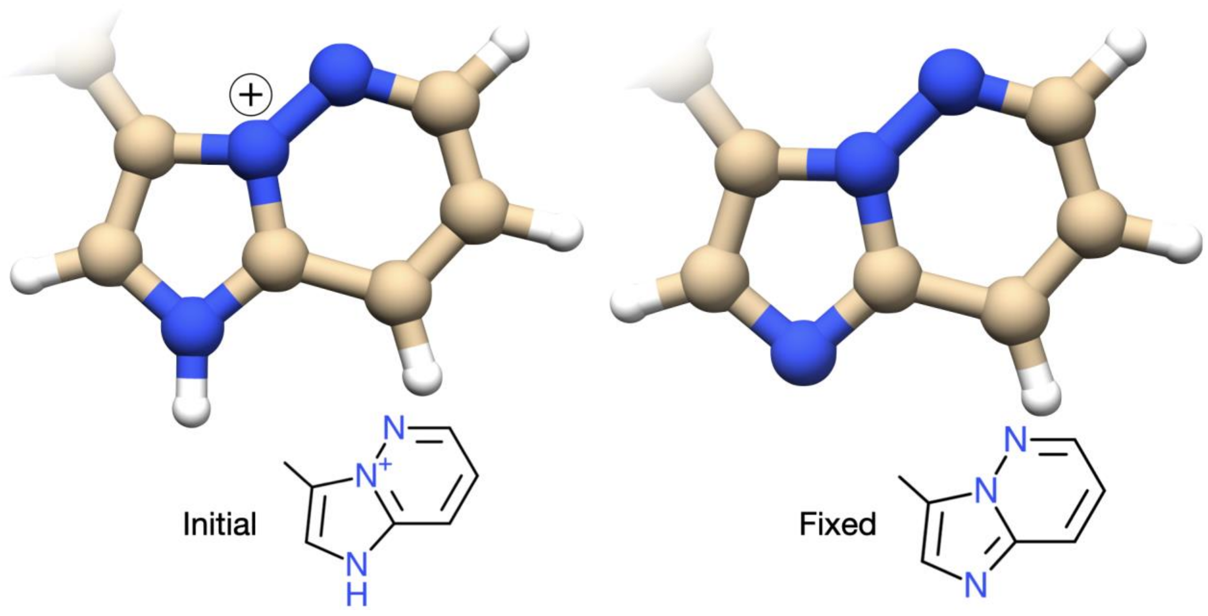

Supplementary Figure 12: Structure of 1PYE.

Original and fixed protonation state for the ligand in the complex 1URW which contains an imidazo-[1,2-b]-pyridazine fragment. We applied the principle of charge neutrality. See 1PYE for another example. Nitrogen (N, blue), Carbon (C, beige), Hydrogen (H, white).

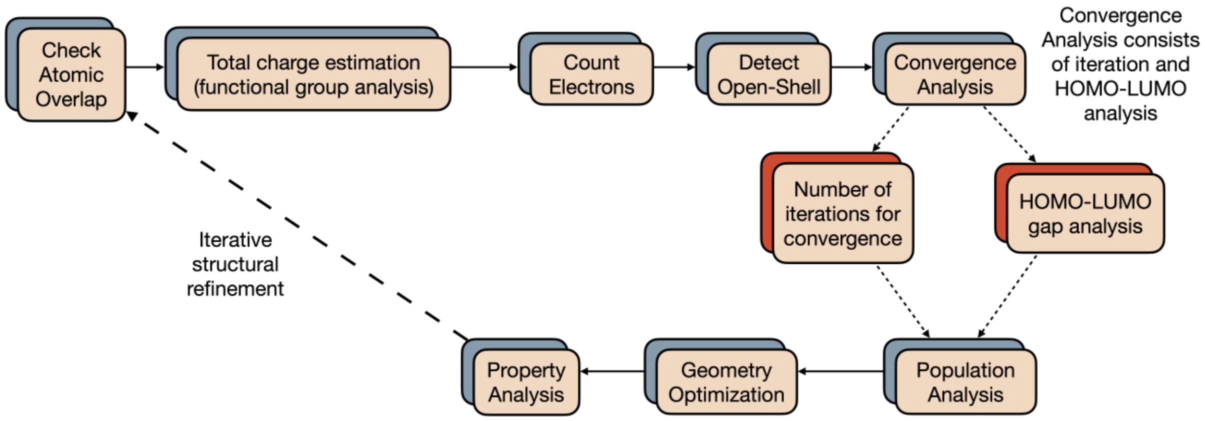

Supplementary Figure 13: **Scheme for structure refinement.**

Main scheme for the protocol used to clean and refine the original structures.

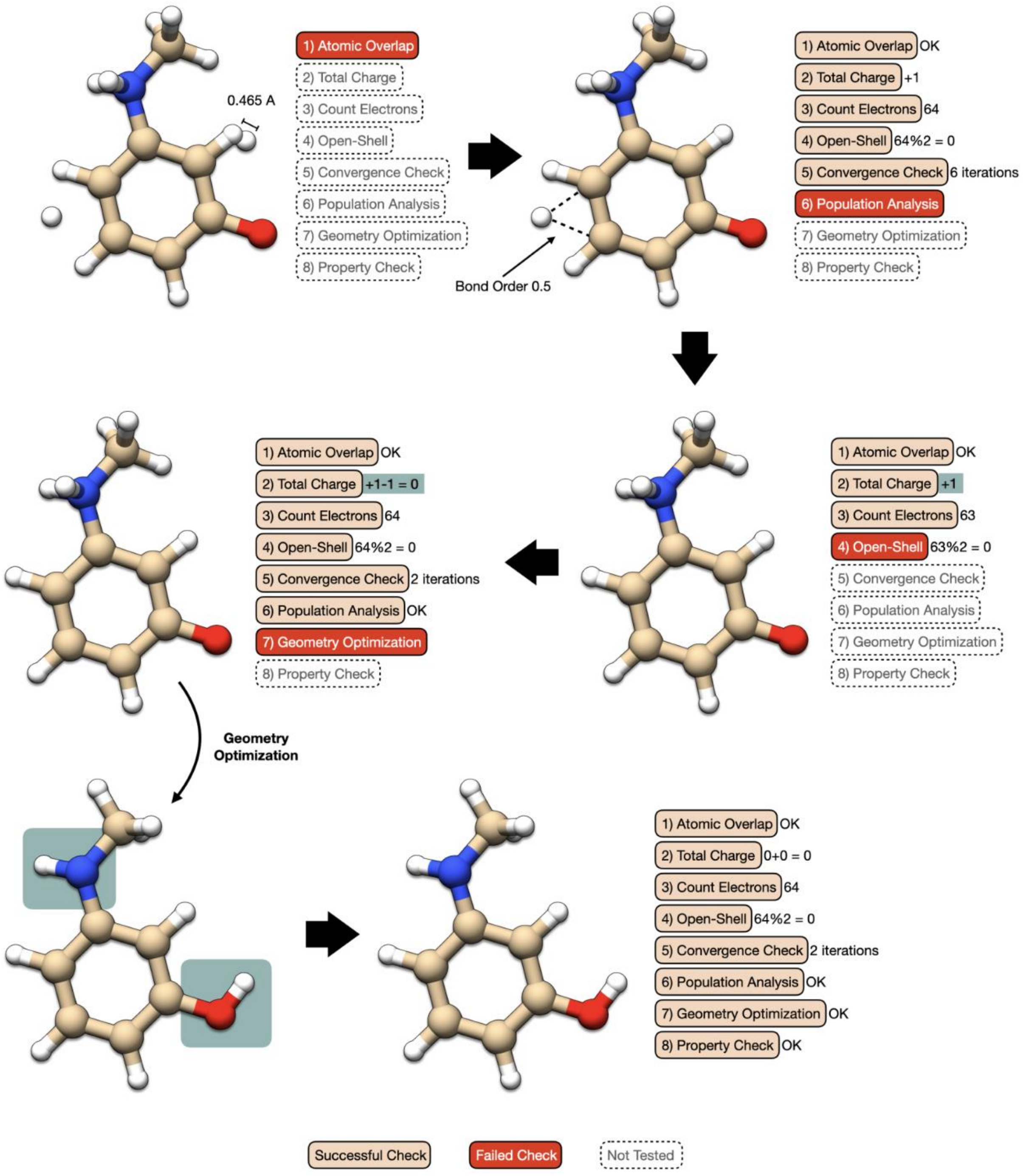

Supplementary Figure 14: Example curation of one molecule.

Example of how the protocol from Supplementary Figure 13 is applied to an example molecule, created to represent some of the problems faced during database curation.

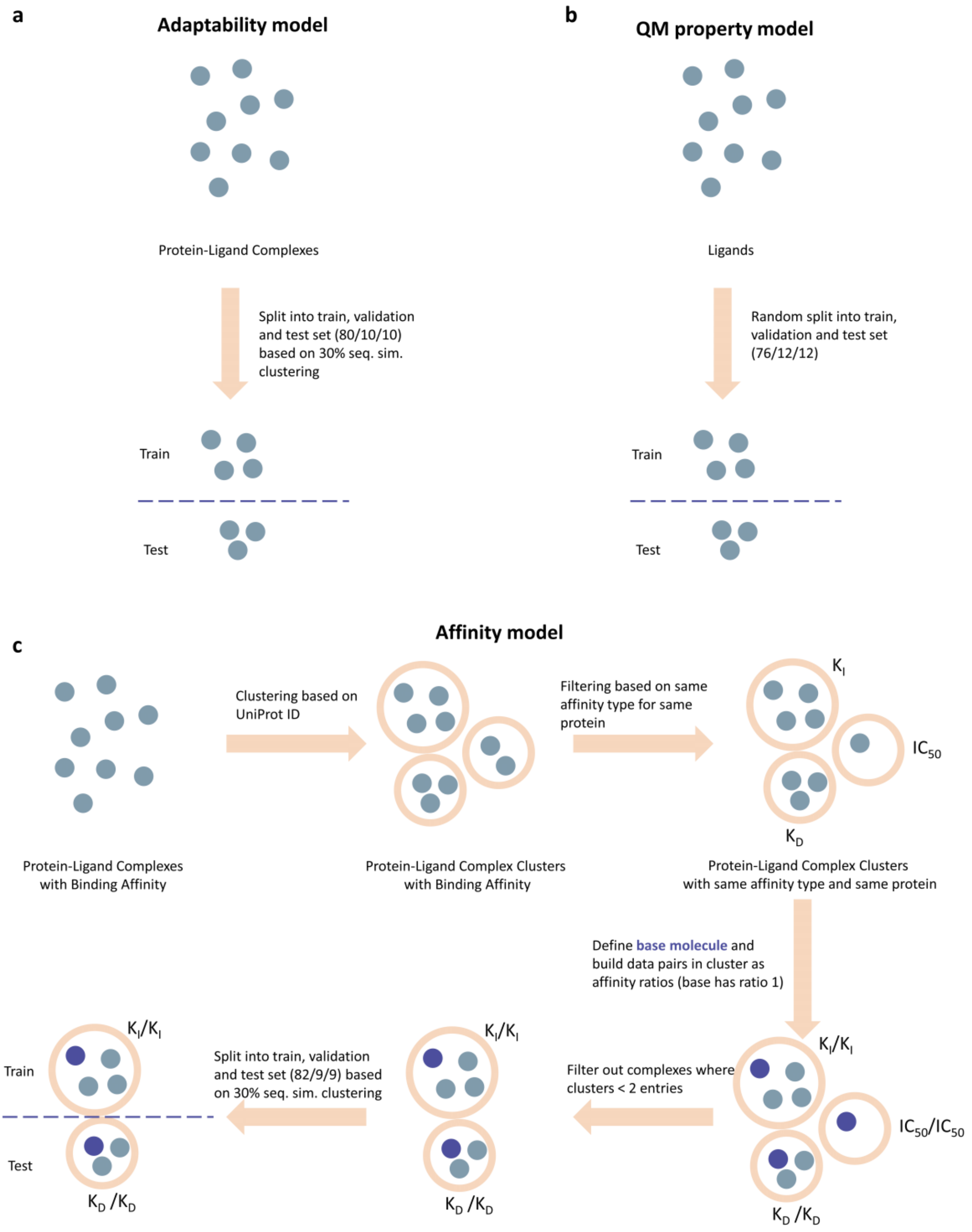

Supplementary Figure 15: Schematic of the data processing workflow for the three baseline models. a, The data for the MD based adaptability model is split into train, validation and test set based on protein sequence similarity. **b,** For the QM property model the splits are performed randomly. **c,** In case of the affinity model the protein-ligand complexes are first clustered based on UniProt ID. These clusters are then divided into subclusters containing the same affinity type. For each of these subclusters a base molecule is defined and clusters with less than 2 entries are filtered out. The splitting of the clusters into train, test and validation is performed based on sequence similarity as for the adaptability model. The exact splits are available via our GitHub repository.

2. Added chemical groups

The list of structures in which we added a chemical group to model effects of covalent binding to the protein are

2FOU, 4JJG, 3CSL, 5WAD, 4XKC, 5OD5, 2P8O, 2Z97, 3W8O, 3RO0, 4Z46, 2FOY, 3ZS1, 2FOV, 5TYJ, 5TYK, 5TYL, 5TYN, 5TYO, 5TYP.

3. Outliers not considered for the QM model

1YHM, 4U6C, 4DZW, 2HAW, 2Z50, 4DXJ, 5IJJ, 2ONB, 4E1E, 2IT4, 2RK8, 2O1C, 3T01, 3C14, 2F89, 2F94, 1A0TB, 1A5G, 4DGO, 4WM9, 6B1X, 1A46, 1A61, 2FSA, 4DWG, 1A0TA, 3BU8, 4UMJ, 3KXZ, 4HZX

4. Evaluation of semi-empirical ionization potentials

Based on the data in the CCCBDB for Koopman ionization potentials, we constructed the table in Supplementary Data 1. We note that though deviations may differ according to functional group, the panorama is generally the same: semi-empirical ionization potentials are of quality comparable to DFT ones, and in some occasions also superior or at least not inferior to MP2 charges. Note that ionization potentials show some dependence on basis set. For fairness in the comparison, we decided to stick to a single basis set of general use by the community of applied theoreticians. We also chose a fair basis set for the evaluation of the property.

5. Heuristics based program for inclusion and processing of new structures

To ease structure processing, a heuristics-based method was included in ULYSSES. This module checks for atomic clashes caused by overly short bonds. Afterwards, the program goes over the atoms in the molecule and checks for the chemical neighbourhood. It then identifies certain patterns, which are associated with chemical groups and their properties. This is used to estimate the total molecular charge. We currently include several classes of functional groups, and more will be added in the future.
The program available from MISATO further includes basic electronic processing of the structures. This includes counting of electrons (with warnings issued if radicals are present), Frontier Molecular Orbital Analysis, bond-order calculation and AM1 charges. The latter may be directly input into programs like Amber.

6. Protocol used for database curation

Supplementary Figure 13 and Supplementary Figure 14 contain a graphical description of the protocol used for database curation. For simplicity, we will provide a detailed description of Supplementary Figure 13, referring to Supplementary Figure 14 when suitable. Note that the protocol is iterative, and it was applied to the whole database, rather than case by case. The protocol starts by looking for short contacts in the molecules. This can be done using several procedures, namely

1) Checking the eigenvalues of the overlap matrix, needed for the quantum mechanical calculations.

2) Calculating all the atomic distances for each system, printing all cases where distances are below a given threshold (e.g., 0.6 Angstrom).

In the example of Supplementary Figure 14, the first iteration shows that two hydrogen atoms are close enough to yield an inconsistency. The least suitable proton has therefore to be removed. After verification that no two atoms are overlapping, the total charge is calculated.

Here we used a topological algorithm that detects and identifies specific atomic patterns, associated with functional groups. Each functional group is then given a formal charge, and the summation of all formal charges yields the estimated total charge. Then we verify whether the system contains unpaired electrons (open-shell character). Though in the second iteration there is no problem arising from counting of electrons, the third iteration shows a system with an odd number of electrons. For these cases, manual inspection took place to fix the total charge and the count of electrons. At this stage, electronic density calculations could take place, so we applied convergence analysis. Here we looked mainly at the number of iterations required for having a stable self-consistent field calculation, and we analyzed in detail systems with small HOMO-LUMO gaps, which could indicate pathologies in the atomic system. With a set of converged electronic densities, we performed population analysis, to count the bond-orders between the atoms. Half-bonds (second iteration of Supplementary Figure 14) or explicit violations of the octet were analyzed in detail, and pathologies fixed. After this check was successful, we assumed that the molecular states were stable and reasonable enough to proceed. We subsequently performed a stability test, where geometry optimization was used to determine whether changes of protonation state, or bond breaking would take place due to the protonation state proposed. Additionally, singlet biradicals could also be identified, as these would lead to an intramolecular reaction. After fixing such inconsistencies, we scouted for outliers in physical properties. An example of one of the tests is provided in Figure 2b of the main text.

7. Example code to access the data

Example code to access QM data:

```
qmh5_file = “../data/QM/h5_files/tiny_qm.hdf5”
qm_H5File = h5py.File(qmh5_file)

# Electron affinity for structure 10GS
qm_H5File[“10GS”][“mol_properties”][“Electron_Affinity”][()]

# Atom’s coordinates for structure 10GS
xyz = qm_H5File[“10GS”][“atom_properties”][“atom_properties_values”][:,0:3]
```

Example code to access MD data:

```
mdh5_file = “../data/MD/h5_files/tiny_md.hdf5”
md_H5File = h5py.File(mdh5_file)

# Interaction energy of the first frame for structure 10GS
interaction_energy = md_H5File[“10GS”][“frames_interaction_energy”][0]

# Atom’s coordinates from the first frame for structure 10GS
xyz = md_H5File[“10GS”][“trajectory_coordinates”][0,:,:]
```
