# Supplementary material for: MISATO: machine learning dataset of protein–ligand complexes for structure-based drug discovery
Source: Nat Comput Sci. 2024 May 10;4(5):367–78. doi: 10.1038/s43588-024-00627-2 (PMC11136668; doi:10.1038/s43588-024-00627-2)
Supplement: Supplementary file 2 — Reporting Summary [file 43588_2024_627_MOESM2_ESM.pdf]

Corresponding author(s): Grzegorz M. Popowicz
Last updated by author(s): Apr 4, 2024

Reporting Summary

Nature Portfolio wishes to improve the reproducibility of the work that we publish. This form provides structure for consistency and transparency in reporting. For further information on Nature Portfolio policies, see our Editorial Policies and the Editorial Policy Checklist.

Statistics

For all statistical analyses, confirm that the following items are present in the figure legend, table legend, main text, or Methods section.

n/a	Confirmed	
☐	☒	The exact sample size (*n*) for each experimental group/condition, given as a discrete number and unit of measurement
☒	☐	A statement on whether measurements were taken from distinct samples or whether the same sample was measured repeatedly
☒	☐	The statistical test(s) used AND whether they are one- or two-sided<br>*Only common tests should be described solely by name; describe more complex techniques in the Methods section.*
☒	☐	A description of all covariates tested
☒	☐	A description of any assumptions or corrections, such as tests of normality and adjustment for multiple comparisons
☐	☒	A full description of the statistical parameters including central tendency (e.g. means) or other basic estimates (e.g. regression coefficient) AND variation (e.g. standard deviation) or associated estimates of uncertainty (e.g. confidence intervals)
☒	☐	For null hypothesis testing, the test statistic (e.g. *F*, *t*, *r*) with confidence intervals, effect sizes, degrees of freedom and *P* value noted<br>*Give P values as exact values whenever suitable.*
☒	☐	For Bayesian analysis, information on the choice of priors and Markov chain Monte Carlo settings
☒	☐	For hierarchical and complex designs, identification of the appropriate level for tests and full reporting of outcomes
☐	☒	Estimates of effect sizes (e.g. Cohen's *d*, Pearson's *r*), indicating how they were calculated

Our web collection on statistics for biologists contains articles on many of the points above.

Software and code

Policy information about availability of computer code

Data collection	Amber20, https://gitlab.com/siriius/ulysses version 1.0.0, Python 3.10.9,
Data analysis	Python 3.10.9, AutoDock Vina 1.2.5, https://github.com/t7morgen/misato-dataset (doi:10.5281/zenodo.10926008)

For manuscripts utilizing custom algorithms or software that are central to the research but not yet described in published literature, software must be made available to editors and reviewers. We strongly encourage code deposition in a community repository (e.g. GitHub). See the Nature Portfolio guidelines for submitting code & software for further information.

Data

Policy information about availability of data

All manuscripts must include a data availability statement. This statement should provide the following information, where applicable:

- Accession codes, unique identifiers, or web links for publicly available datasets
- A description of any restrictions on data availability
- For clinical datasets or third party data, please ensure that the statement adheres to our policy

MISATO is publicly accessible and can be downloaded from Zenodo (https://zenodo.org/records/7711953, DOI: 10.5281/zenodo.7711953). We provide instructions for usage, data loaders via our GitHub repository, and a container image with all relevant packages installed for GPU usage. Source data of Figures 2,3,5,6 is available with this manuscript. MISATO was built from PDBbind database (release 2022).

Human research participants

Policy information about studies involving human research participants and Sex and Gender in Research.

Reporting on sex and gender	Not applicable
Population characteristics	Not applicable
Recruitment	Not applicable
Ethics oversight	Not applicable

Note that full information on the approval of the study protocol must also be provided in the manuscript.

Field-specific reporting

Please select the one below that is the best fit for your research. If you are not sure, read the appropriate sections before making your selection.

☒ Life sciences ☐ Behavioural & social sciences ☐ Ecological, evolutionary & environmental sciences

For a reference copy of the document with all sections, see nature.com/documents/nr-reporting-summary-flat.pdf

Life sciences study design

All studies must disclose on these points even when the disclosure is negative.

Sample size	Study was performed on a set of ~20000 published molecular structures. Entire public repository was used for the study.
Data exclusions	For MD structures were disregarded whenever non-standard ligand atoms (Metal ions) or inconsistencies in the protein starting structures were encountered. For QM ML model a small number (30) of structures were omitted due to inability of current algorithm to provide correct predictions for them. This does not introduce a bias to the observation and does not change our observations.
Replication	Besides NMR no experimental work has been conducted in this manuscript. As a standard, NMR experiments are not performed in replicates. This is because they rely on averaging and Fourier transformation of huge number of experimental samples internally and the measurement error is not arising from single point measurement. The statistical error of NMR experiments is intrinsically captured within one experiment and dominates the error value, the systematic error of NMR measurements is considered very low.
Randomization	The splits for train, test and validation were randomized for the different ML models. The exact procedure for each model is given in Supplementary Figure S15. The data for the MD based adaptability model is split into train, validation and test set based on protein sequence similarity. For the QM property model the splits are performed randomly. In case of the affinity model the protein-ligand complexes are first clustered based on UniProt ID. These clusters are then divided into subclusters containing the same affinity type. For each of these subclusters a base molecule is defined and clusters with less than 2 entries are filtered out. The splitting of the clusters into train, test and validation is performed based on sequence similarity as for the adaptability model. The exact splits are available via our GitHub repository.
Blinding	No blinding was used in our study. The data we worked with was objective, large and not subject to interpretation or bias. Moreover, we used algorithmic processing of the data that worked uniformly on all data with objective metrics.

Reporting for specific materials, systems and methods

We require information from authors about some types of materials, experimental systems and methods used in many studies. Here, indicate whether each material, system or method listed is relevant to your study. If you are not sure if a list item applies to your research, read the appropriate section before selecting a response.

Materials & experimental systems

n/a	Involved in the study
☒	☐ Antibodies
☒	☐ Eukaryotic cell lines
☒	☐ Palaeontology and archaeology
☒	☐ Animals and other organisms
☒	☐ Clinical data
☒	☐ Dual use research of concern

Methods

n/a	Involved in the study
☒	☐ ChIP-seq
☒	☐ Flow cytometry
☒	☐ MRI-based neuroimaging
